# Warfarin and Flavonoids Do Not Share the Same Binding Region in Binding to the IIA Subdomain of Human Serum Albumin

**DOI:** 10.3390/molecules22071153

**Published:** 2017-07-11

**Authors:** Hrvoje Rimac, Claire Dufour, Željko Debeljak, Branka Zorc, Mirza Bojić

**Affiliations:** 1 Department of Medicinal Chemistry, University of Zagreb, Faculty of Pharmacy and Biochemistry, Ante Kovačića 1, 10000 Zagreb, Croatia; hrimac@pharma.hr (H.R.); bzorc@pharma.hr (B.Z.); 2 UMR408 SQPOV, Safety and Quality of Plant Products, INRA, Avignon University, 228 Route de l’Aérodrome, 84000 Avignon, France; claire.dufour@inra.fr; 3 Institute of Clinical Laboratory Diagnostics, Osijek University Hospital Center, Josipa Huttlera 4, 31000 Osijek, Croatia; debeljak.zeljko@kbo.hr; 4 Department of Pharmacology, School of Medicine, University of Osijek, Cara Hadrijana 10/E, 31000 Osijek, Croatia

**Keywords:** flavonoids, HSA binding, subdomain IIA, fluorimetry, docking, molecular modeling

## Abstract

Human serum albumin (HSA) binds a variety of xenobiotics, including flavonoids and warfarin. The binding of another ligand to the IIA binding site on HSA can cause warfarin displacement and potentially the elevation of its free concentration in blood. Studies dealing with flavonoid-induced warfarin displacement from HSA provided controversial results: estimated risk of displacement ranged from none to serious. To resolve these controversies, in vitro study of simultaneous binding of warfarin and eight different flavonoid aglycons and glycosides to HSA was carried out by fluorescence spectroscopy as well as molecular docking. Results show that warfarin and flavonoids do not share the same binding region in binding to HSA. Interactions were only observed at high warfarin concentrations not attainable under recommended dosing regimes. Docking experiments show that flavonoid aglycons and glycosides do not bind at warfarin high affinity sites, but rather to different regions within the IIA HSA subdomain. Thus, the risk of clinically significant warfarin–flavonoid interaction in binding to HSA should be regarded as negligible.

## 1. Introduction

Human serum albumin (HSA) is a plasma protein capable of binding and transporting a wide variety of ligands, both endogenous and exogenous. It serves as a depot for xenobiotics, prolonging their half-life in circulation and regulating their blood concentration [1]. HSA is a protein with a molar mass of 66,500 g/mol and its blood concentration in healthy subjects ranges from 500–750 μM (35–50 g/L) [2]. It consists of three domains (I–III), each of which is comprised of two subdomains (A and B) and the two most important binding sites for drugs are situated in subdomains IIA and IIIA, which are also called warfarin and benzodiazepine site, respectively [3].

A large number of drugs characterized by a narrow therapeutic range, one of which is warfarin, binds to HSA [4]. The upper limit of therapeutic range for warfarin is approximately 9 μM (3 mg/L) [5,6]. Since most of warfarin molecules in blood are bound to HSA (>99%) [7] the presence of another compound which also binds to HSA can theoretically cause displacement of warfarin leading to elevation of free warfarin concentration [8,9]. These interactions could lead to adverse effects in patients suffering from a kidney or liver disease [10,11,12]. Although some of the aforementioned in vitro data implies otherwise, displacement of warfarin from HSA is regarded as clinically insignificant under the normal dosing regime [11,13].

Flavonoids are plant products abundant in every day diet [14]. They have many salutary effects, of which their antioxidative [15], cardiovascular [16,17], and anticarcinogenic [18] are the most extensively studied. In some instances, their plasma concentrations may rise to micromolar values, e.g., after consumption of high quantities of onions, green tea, orange juice etc. [17,19,20]. They generally bind to the IIA site of HSA [21,22], suggesting that their binding may cause displacement of drugs from these binding sites, leading to adverse effects [23,24]. Once in circulation, flavonoids are extensively conjugated, mostly by glucuronidation [25] and sulfatation [26]. This is of critical importance for appropriate assessment of possible flavonoid interactions with other endogenous or exogenous ligands.

**Figure 1:**
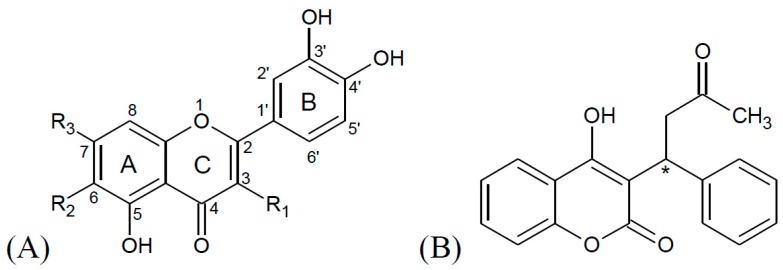
Chemical structures of (**A**) investigated flavonoids and (**B**) warfarin. See Table 1 for individual flavonoids.

Recent research about flavonoid-induced displacement of warfarin from HSA produced controversial results [21,23,24,27,28]. Several different methods were used, including fluorescence spectroscopy [21,24], circular dichroism [23,28], nuclear magnetic resonance [23], ultracentrifugation [27], electronic absorption spectroscopy and molecular modeling [28], but the results vary from no risk of displacement [21] to significant risk of adverse effects associated with warfarin [24]. In this research, simultaneous binding of warfarin and different flavonoids (both aglycons and glycosides) (Figure 1) to the IIA HSA binding site was studied. Binding of ligands to the IIA binding site of HSA can easily be monitored by fluorescence spectroscopy [29,30]. This binding site is characterized by a hydrophobic cavity and a hydrophilic, positively charged entrance, ideal conditions for binding of small, negatively charged ligands such as warfarin or flavonoids [31]. A well-known property of this binding site is the presence of a single tryptophan residue (Trp-214), which fluoresces in aqueous solutions. When a ligand binds in the tryptophan vicinity, the fluorescence intensity decreases. This property enables determination of the ligand binding constant as well as calculation of the distance from the tryptophan residue [29,30]. Additionally, when unbound in a solution, warfarin and certain flavonoids are slightly fluorescent, but when they are bound to HSA, the intensity of their fluorescence increases considerably [21,32], making it possible to monitor their binding or displacement.

Displacement studies were conducted at excitation and emission wavelengths of warfarin and flavonoids. Quercetin-3-*O*-glucuronide was included in this study as a representative of flavonoid glucuronides. Docking calculations for the HSA-quercetin and the HSA-quercetin-3-*O*-glucuronide complexes were also performed to improve the interpretation of in vitro observations and to determine their precise binding locations on HSA, as well as their interaction with a putative second, low affinity warfarin binding site.

## 2. Results and Discussion

### 2.1. Ligand Binding Constants

Quenching of the tryptophan fluorescence can be used to determine binding constants of ligands which bind in this region of HSA. Results are shown in Table 1.

Warfarin and flavonoids are derivatives of benzopyranone (Figure 1), so it may be hypothesized that they share the same binding site on HSA. Among the investigated ligands, quercetin is characterized by the highest binding constant, followed by luteolin and warfarin (Table 1). Lower values of binding constants for glycosides can be explained by their size. This is shown by rutin which, as the largest tested glycoside, has the lowest binding constant. The negative charge of the glucuronic acid moiety of quercetin-3-*O*-glucuronide slightly increases its binding constant compared to other glycosides, consistent with the premise about the strong binding of anions to IIA binding site [31,33].

### 2.2. Warfarin Displacement Studies

In order to evaluate the risk of drug displacement from HSA, in vitro techniques involving low HSA concentrations are usually employed [8,34,35]. Results obtained in this way cannot be directly extrapolated to physiological conditions, but can provide valuable insights [36]. For warfarin displacement studies, flavonoid fluorescence was used. All of the tested flavonoids have the 5-OH group, which is known to quench their intrinsic fluorescence [32]. However, when bound to HSA, these flavonoids fluoresce at excitation and emission wavelengths of 450 and 500–540 nm, respectively. If a flavonoid is unable to bind to HSA or binds with a lower or higher constant due to an external factor, this can be observed from the intensity of their maximum fluorescence (*F*_max_) and the concentration needed to achieve it (*c*_max_). With the binding constant of warfarin for HSA calculated to be (10.56 ± 0.68) × 10^4^ M^−1^ and by application of Equations (4) and (5), it is possible to determine warfarin concentration needed to saturate a certain fraction of HSA binding sites, with presumed 1:1 binding ratio. It has to be pointed out that the warfarin sodium used in this study was racemic mixture: the binding constants for (*R*)- and (*S*)-warfarin are not identical, with the (*S*)-enantiomer having a slightly higher binding constant [37]. However, as already stated, they do bind to the exact same region of the IIA subdomain [38].

#### 2.2.1. Simultaneous Binding of Warfarin and Flavonoid Aglycons

**Figure 2:**
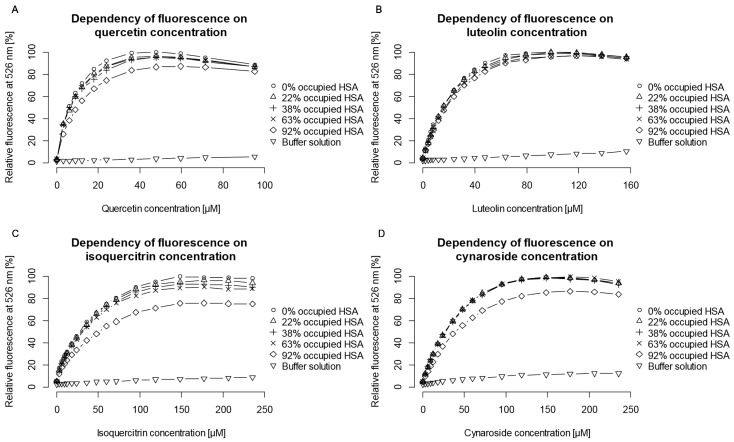
Effect of different warfarin concentrations on fluorescence of human serum albumin (HSA)-flavonoid complexes. (**A**) Quercetin; (**B**) luteolin; (**C**) isoquercitrin; and (**D**) cymaroside.

After incubation with warfarin, flavonoid concentration was gradually increased until *F*_max_ was achieved. Warfarin displacement due to its simultaneous binding to HSA with quercetin, luteolin, isoquercitrin, or cynaroside is represented by changes in fluorescence (Figure 2). The fact that some flavonoid aglycons bind more firmly to HSA in comparison to warfarin can suggest consequential warfarin displacement. The determination of the warfarin binding constant enabled calculation of the concentration needed to saturate a desired fraction of the IIA binding site. In such cases, obstruction of flavonoid binding will also decrease their fluorescence.

**Figure 3:**
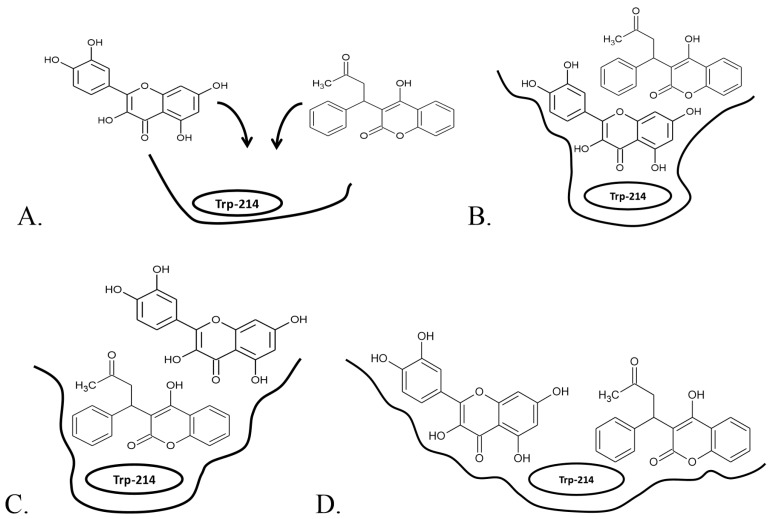
Proposed locations of warfarin and quercetin molecules in the IIA site of HSA. (Based on Petitpas et al. [38] and Ghuman et al. [44]). (**A**) Warfarin and quercetin bind to the same binding region; (**B**) warfarin binding region is situated in front of the quercetin binding region; (**C**) warfarin binding region is situated behind quercetin binding region; (**D**) warfarin and quercetin binding regions are independent of each other.

Addition of warfarin slightly decreased quercetin *F*_max_ with a slight increase in *c*_max_ (Figure 2A). A decrease of quercetin *F*_max_ with increasing warfarin concentration was already established for a bovine serum albumin (BSA)-quercetin complex [39]. This decrease is most evident when 92% of the IIA binding site is saturated with warfarin. In this case warfarin concentration is 120 μM, as opposed to a quercetin concentration of less than 60 μM, which is much higher than physiologically obtainable concentrations. For lower values of HSA saturation there is no significant difference in either *F*_max_ or *c*_max_. It can be concluded that warfarin and quercetin do not share the same binding region even though they are both located in the IIA subdomain (Figure 3A). If the quercetin binding region would be situated farther inside the binding pocket than that of warfarin (Figure 3B), warfarin would impede quercetin binding and at high warfarin concentrations, the fluorescence at 526 nm would be quenched completely. However, these results, supported by docking experiments, do not confirm this scenario. Additional experiments (see Table S1 and Figure S1) were conducted where HSA was saturated with quercetin and warfarin concentration was gradually increased to determine if warfarin binding region is located farther inside the binding pocket than quercetin (Figure 3C). These results also support the hypothesis that there are no direct interactions between quercetin and warfarin. In both cases, even at the highest concentrations of the obstructing ligand, a small addition of the tested ligand causes significant rise in fluorescence intensity. Thus, warfarin and quercetin neither share the same binding region, nor is the binding region of one ligand situated in front of the other (Figure 3D), but rather a formation of a less fluorescent ternary HSA-quercetin-warfarin complex occurs, where quercetin and warfarin bind in the same, IIA binding domain, but in different regions and do not significantly influence each other’s binding affinity. This is further confirmed by a lack of distinctive sigmoidal or Langmuir-like curves in cases of cooperative [40,41] or anti-cooperative binding [42,43], respectively (Figure 2). Similar results were obtained for luteolin (Figure 2B): the only difference is that there was no noticeable change in *F*_max_ and *c*_max_ between different percentages of saturated HSA, even in the case of 92% HSA saturation. It can be concluded that the 3-OH substituent is of high significance for fluorescence intensity of flavonoid molecules, and its position highly influences flavonoid *F*_max_. Another explanation could be the presence of warfarin low affinity site (LAS), which could potentially explain its higher impact on quercetin than on luteolin molecule as quercetin has an extra 3-OH group that could interact with warfarin bound to LAS at higher concentrations.

Our results—that flavonoids and warfarin do not share the same binding region—are consistent with results published by Zsila et al. [28] and Dufour and Dangles [21]. Further support comes from crystallographic structures obtained by Petitpas et al. [38] and research done by Yamasaki et al. [43,45] stating that the IIA binding site can accommodate additional ligands in the warfarin vicinity. The difference in flavonoid fluorescence can be attributed to conformational and possible size changes of the IIA binding site caused by warfarin. This causes changes in flavonoid binding site and flavonoid *F*_max_, without a significant impact on their binding constant. Difference in flavonoid fluorescence at 92% HSA saturation can be explained by a more pronounced conformational change of the binding site, but the possibility of a secondary warfarin molecule bound at its LAS at higher warfarin concentrations directly influencing flavonoid fluorescence intensity cannot be excluded [27].

Results published by Di Bari et al. [23] and Poór et al. [24,46] state that quercetin, as well as some other flavonoids, can displace warfarin from its binding site even at lower concentrations. Di Bari et al. stated that the decrease in intensities of quercetin induced circular dichroism (ICD) bands suggests its displacement from HSA. Additionally, Poór et al. stated that the decrease of polarization values corresponds to higher rotational freedom of the free drug, i.e., displacement of warfarin from its albumin complex. As shown by Yamasaki et al. [43,45], binding of a second ligand can influence the mobility of the first ligand and affect ICD and fluorescence anisotropy without displacement involved; changes in fluorescence anisotropy or induced circular dichroism do not unequivocally suggest ligand displacement. Additionally, the saturation transfer difference nuclear magnetic resonance (STD-NMR) technique used by Di Bari et al. is characterized by the dependence of signal intensity on the proximity of ligand hydrogen atoms to HSA hydrogen atoms. Signal intensity depends on the inverse sixth power of the ligand-HSA distance and the relative signal intensity of quercetin in presence of warfarin was ~40% of its intensity without warfarin. Therefore, binding of warfarin does influence the distance between quercetin and HSA, but the results do not explicitly show displacement. They can also suggest conformational changes.

#### 2.2.2. Simultaneous Binding of Warfarin and Flavonoid Glycosides

Additional measurements were performed under the assumption that warfarin would obstruct binding of flavonoid glycosides more easily. Flavonoid glycosides have lower binding constants and are bulkier so they are expected to interact with warfarin more easily than their aglycon counterparts. As with aglycons, two different behaviors were observed: (a) quercetin derivatives (all substituted at position 3) experience a slight decrease in *F*_max_ with little or no impact on the *c*_max_, except with HSA being 92% saturated (Figure 2C, data for the other quercetin derivatives are not shown due to similarity) and (b) luteolin derivatives (substituted at positions 6 and 7) experience no decrease in *F*_max_ nor *c*_max_, again with the exception of the HSA being 92% saturated (Figure 2D, data for isoorientin are not shown due to similarity). Substituents at position 3 influence the fluorescence intensity as they participate in the large conjugated system connecting the B ring with the 4-oxo group, but have almost no effect on the interactions with warfarin. Substitution at positions 6 or 7 has no effect on flavonoid fluorescence nor on interactions with warfarin. As with quercetin, the only interaction observed was at 92% HSA saturation. This can be explained by the binding of warfarin to a LAS [38,39,44] causing allosteric interactions or energy transfer with flavonoids and formation of a less fluorescent ternary complex or by a direct competitive interaction. Also, as with quercetin and luteolin, it is evident that warfarin has a higher influence on quercetin than on luteolin glycosides, emphasizing the influence of the 3-OH group on fluorescence intensity.

### 2.3. Molecular Modeling of the Quercetin-HSA and Quercetin-3-O-glucuronide-HSA Complexes

**Figure 4:**
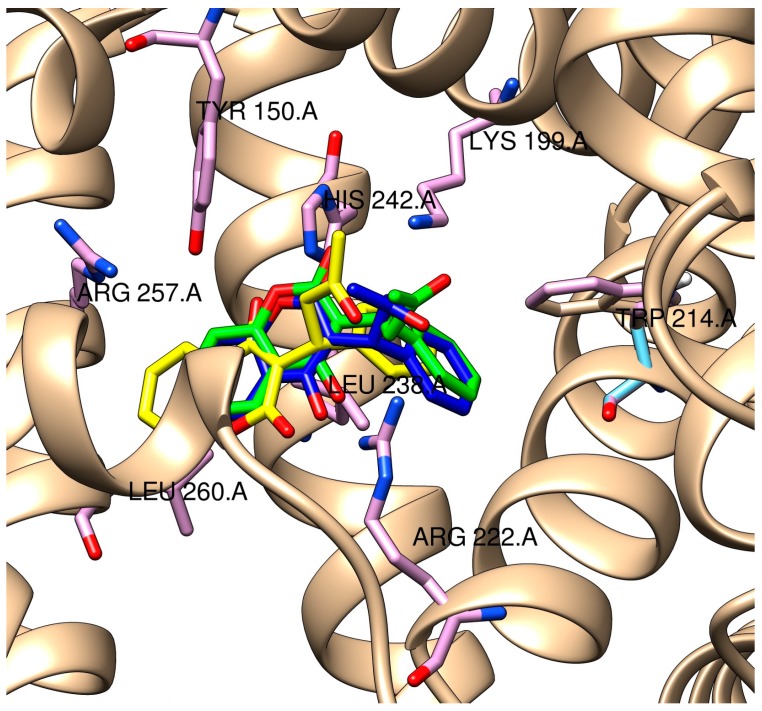
Overlay of crystallographic structure of (*R*)-warfarin (yellow) with docked (*R*)-warfarin (blue) and (*S*)-warfarin (green).

The comparison of docked (*R*)- and (*S*)-warfarin with the crystallographic structure of (*R*)-warfarin bound to HSA (Protein Data Bank (PDB) entry 2BXD) showed high similarity of ligands’ positions (Figure 4) and validating the appropriateness of this approach. In warfarin vicinity, there are four positively charged residues, Lys-199, Arg-222, His-242, and Arg-257, and three residues that are not charged, Tyr-150, Leu-238, and Leu-260. Warfarin also forms three hydrogen bonds, with Tyr-150, Arg-222, and His-242.

**Figure 5:**
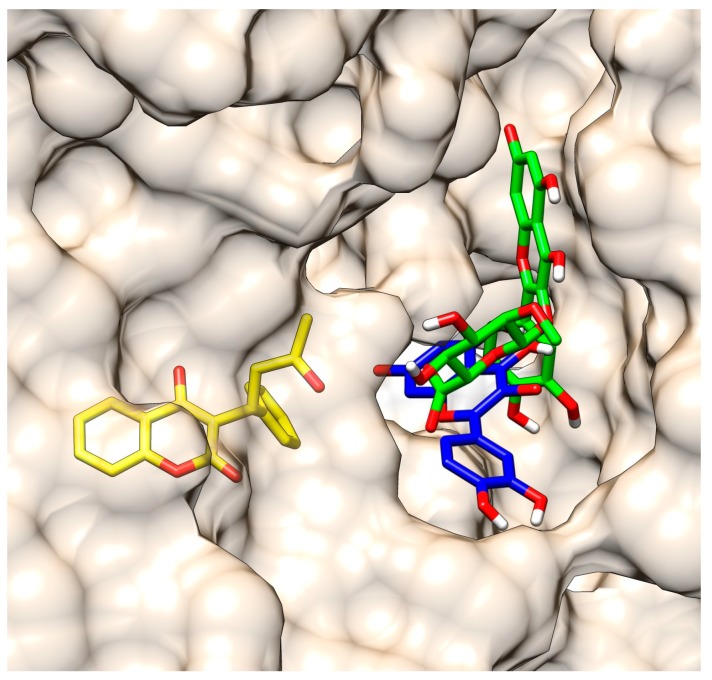
Overlay of crystallographic structure of (*R*)-warfarin (yellow) with docked fluorescent species of quercetin (blue) and quercetin-3-*O*-glucuronide (green).

A docking summary for quercetin species can be found in Table 2. From all the shown clusters, only clusters number 1 and 3 of both 7- and 4′-quercetin anions and cluster 4 from 3,4′-fluorescent quercetin anion show overlap in binding positions with warfarin, but these clusters are formed from a very low number of runs (3 and 2 for quercetin anion at position 7, 1 and 1 for quercetin anion at position 4′, and 3 for fluorescent quercetin anion at positions 3 and 4′, respectively) and thus are unlikely. For all the other runs, for both non-fluorescent and fluorescent species, quercetin binding site does not overlap with that of warfarin and is located at the hydrophilic entrance of the IIA subdomain (Figure 5).

**Figure 6:**
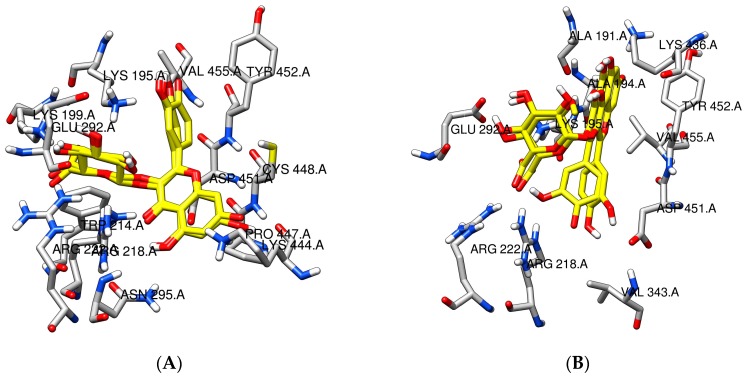
Comparison of docked positions of quercetin-3-*O*-glucuronide species (yellow) with nearby amino acid residues. (**A**) quercetin-3-*O*-glucuronide anionic species; (**B**) fluorescent quercetin-3-*O*-glucuronide anionic species. Nitrogen atoms are colored blue, oxygen atoms red, hydrogen atoms white, and HSA carbon atoms grey.

On the other hand, the binding site of quercetin-3-*O*-glucuronide does not overlap with the binding site of warfarin in any of the runs, but overlaps with the quercetin binding site. The number of runs per cluster for quercetin-3-*O*-glucuronide (Table 3) is lower than that of quercetin, which can be explained by a greater flexibility of the glucuronide ligand, as well as by the spaciousness of the HSA IIA entrance which is able to accommodate it in various conformations. The binding sites of all the clusters is located at the mouth of the IIA subdomain (Figure 5), with the aglycon moiety of anionic and fluorescent anionic species being rotated by approximately 180°. The glucuronic moiety of the molecules is located in the same plane, but in the case of fluorescent anionic species, it is rotated by 90° and protrudes towards the protein surface (Figure 6), which can be the cause of weaker binding (Table 3).

**Figure 7:**
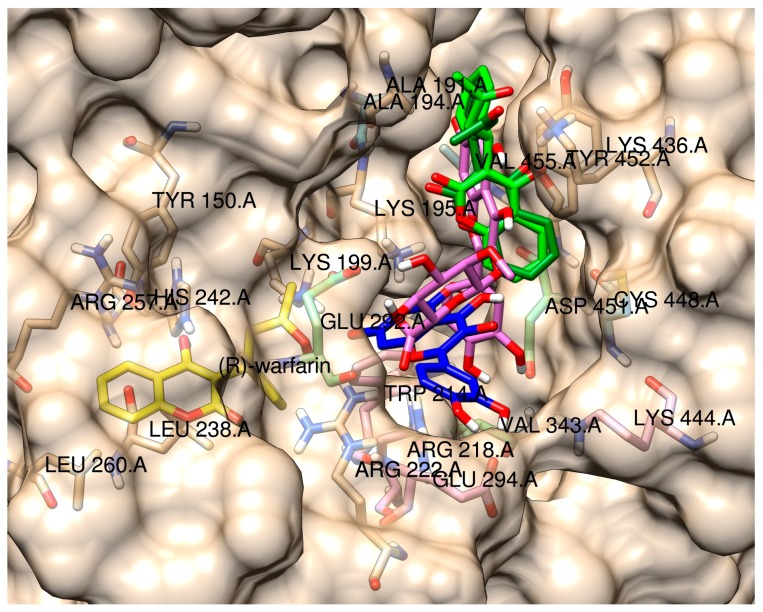
Comparison of crystallographic data of (*R*)-warfarin (yellow) with docked position of (*R*)- and (*S*)-warfarin at LAS (both green), fluorescent quercetin anion at positions 3 and 7 (blue), and fluorescent quercetin-3-*O*-glucuronide anion at position 7 (pink) with nearby amino acid residues.

Docking results of (*R*)- and (*S*)-warfarin in order to locate warfarin’s LAS (Table 4) both showed (almost unanimously for all runs for both isomers) a high preference for a binding region located in the vicinity of Ala-191, Lys-195, and Tyr-452, which are amino residues that also interact with flavonoid glycosides in their binding to HSA but do not have high significance in flavonoid aglycone binding (Figure 7). From the data, it can be concluded that warfarin binding to its LAS could compete with flavonoid glycosides in binding to HSA as the location of the coumarin and flavonoid AC rings overlap. This can explain lower fluorescence of flavonoid glycosides at high warfarin concentrations. On the other hand, warfarin bound to its LAS does not seem to interfere with binding of flavonoid aglycons. However, the proximity of the conjugated AC rings (see Figure 1) to the warfarin coumarin ring could explain lower quercetin fluorescence at high warfarin concentrations via energy transfer. Luteolin is less affected by this energy transfer in terms of relative change in fluorescence intensity (Figure 2). However, its absolute fluorescence is lower in all cases precisely due to the lack of the 3-OH group (see Table S2).

## 3. Materials and Methods

### 3.1. Chemicals and Materials

Fatty acid free HSA and warfarin sodium were purchased from Sigma-Aldrich (St. Louis, MO, USA). Flavonoids were obtained from Sigma-Aldrich (quercetin), TransMIT GmbH (Gießen, Germany) (isoquercitrin, rutin, hyperoside, cynaroside, and isoorientin), and Extrasynthèse (Genay, France) (luteolin and quercetin-3-*O*-glucuronide). All standards had a specified purity of ≥98% and have been used without further purification.

### 3.2. Fluorescence Measurements

HSA was freshly dissolved in Dulbecco’s phosphate-buffered saline pH 7.4 (137 mM sodium chloride, 2.7 mM potassium chloride, 8.1 mM disodium hydrogen phosphate, 1.47 mM potassium dihydrogen phosphate) immediately before measurements. The stock solution of warfarin sodium was prepared in MeOH, while the stock solution of luteolin was prepared in MeOH:DMSO = 2:1, and of other flavonoids in MeOH:DMSO = 4:1. In all experiments, the final co-solvent concentration was held below 5%. This was done to prevent conformational changes of HSA due to changes in dielectric constant of the solvent.

Steady-state fluorescence was recorded using a thermostated Safas Xenius fluorometer (Safas Monaco, Monaco) with an integrated stirrer. All studies were performed at 25 ± 1 °C using 10 nm excitation and emission slit widths. Warfarin and flavonoid solutions were added to a 2 mL of a 6 μM (0.4 g/L) HSA solution placed in a quartz cell. Higher concentrations of HSA and ligands were not used due to poor solubility of ligands and high influence of ligand absorption. Measurements were performed in duplicate.

### 3.3. Determination of Binding Constants

A stock solution of a single ligand was gradually added via syringe to a 2 mL of a 6 μM HSA solution placed in a quartz cell. Measurements were conducted at the excitation wavelength of 295 nm and emission was recorded in the 310–400 nm range enabling calculations of corresponding binding constants. As flavonoids and warfarin both absorb light at 295 and 320 nm, their molar absorption coefficients (ε) were calculated at these wavelengths using a water-thermostated HP 8453 UV-visible spectrophotometer (Agilent Technologies Inc., Santa Clara, CA, USA) at 25 ± 0.1 °C to take into account the inner filter effect. Binding constants were determined according to Dufour and Dangles [21]. A Scatchard analysis was performed assuming *n* identical binding sites on HSA. Values for binding constant (*K*), molar fluorescence intensity of the complex (*f*), and the stoichiometric coefficient (*n*) were estimated by fitting the F vs. Lt curves against Equations (1) and (2) using a least-square regression program Scientist (MicroMath, Salt Lake City, UT, USA), where *F* is fluorescence intensity, [*L*] the free ligand concentration, [*L*_t_] the total ligand concentration, and c the total albumin concentration. Since all values of n were close to 1, a simple 1:1 binding model was used. In all cases, the inner filter effect was taken into account by using absorption of free ligands at excitation and emission wavelengths (ε_ex_ and ε_em_, respectively) (Equation (3)), where *F*_corr_ is the fluorescence corrected for the inner filter effect, *F*_0_ is the measured fluorescence, ε is the sum of ε_ex_ and ε_em_, *l* = 0.65 is the optical path and *c* is the flavonoid concentration.

(1) $$F_{corr}=fc\frac{nK\left[L\right]}{1+K\left[L\right]}$$

(2) $$[L_{t}]=\left[L\right]\left(1+\frac{ncK}{1+K\left[L\right]}\right)$$

(3) $$F_{0}=F_{corr}\times 10^{-\varepsilon lc}$$

### 3.4. Fluorescence Measurements

In all experiments, the concentration of HSA was also kept at 6 μM while the fraction of HSA saturated with warfarin has been varied: 0%, 22%, 38%, 63% and 92%. The saturated fraction was calculated using the Equations (4) and (5),

(4) $$K=\frac{\left[cL\right]}{\left[c_{f}\right]\left[L\right]}$$

(5) $$p=\frac{\left[cL\right]}{\left[c\right]}$$

where *K* is the binding constant, [*cL*] is the concentration of HSA-ligand complex, [*c_f_*] is the concentration of unbound HSA, [*L*] is the free ligand concentration, [*c*] is the total HSA concentration and *p* is the fraction of saturated HSA. Flavonoid binding measurements were conducted at the excitation wavelength of 450 nm to maximize the fluorescence of the bound flavonoid and emission was recorded in the 466–580 nm range. Competition of warfarin and flavonoids for binding to HSA was determined based on the maximum intensity of fluorescence (*F*_max_) of flavonoids and the concentration needed to achieve it (*c*_max_). Additional measurements were taken at the excitation and emission wavelengths of warfarin, 317 and 380 nm, respectively, to confirm data obtained from the flavonoid fluorescence measurements.

### 3.5. Docking Experiments

The AutoDock 4.2.6. (The Scripps Research Institute, La Jolla, CA, USA) [47] uses dispersion, hydrogen bonding, and electrostatic and desolvatation energy components to determine the conformation of the most probable complex. It was used to locate probable quercetin, quercetin-3-*O*-glucuronide, and secondary (*R*)- and (*S*)-warfarin binding sites on the HSA molecule. Quercetin was chosen for these experiments as a representative of flavonoid aglycons, and quercetin-3-*O*-glucuronide was chosen as a representative of quercetin metabolites. The three-dimensional coordinates of HSA molecule co-crystallized with (*R*)-warfarin was obtained from the RCSB [44]. This structure was chosen because it is the only one where HSA was crystallized only with warfarin (without myristic acid) and best suits our experimental conditions. Monomer A was selected for the docking procedure and missing side-chain atoms were added. The warfarin molecule, as well as water molecules, were removed from the file used for docking of flavonoids. Hydrogens were added to the HSA molecule, all Lys, Arg, and Cys side-chains were protonated, all Asp and Glu side-chains were deprotonated, the amino and carboxy termini were charged, the His-242 side-chain was *N*_ε_-protonated, and all other His side-chains were *N*_δ_-protonated based on visual inspection, resulting in the net charge of -14. Due to their similarity to flavonoids, docking of (*R*)- and (*S*)-warfarin was also performed and their docking locations were determined to be the same and correspond to the location of the crystallized (*R*)-warfarin. This is in correspondence with previous research showing that (*R*)- and (*S*)-warfarin bind at the same region of Sudlow’s site I. [35] Additionally, docking of (*R*)- and (*S*)-warfarin was also performed in presence of crystallized (*R*)-warfarin to locate a putative secondary binding site located in Sudlow’s site I. The three-dimensional forms of the ligands were drawn and their initial geometries were optimized in HyperChem 8.0 (Hypercube, Inc., Gainesville, FL, USA), and their charge was set to represent the most abundant species at pH 7.4, calculated according to Rimac et al. [48], as well as verified at chemicalize.com. For (*R*)- and (*S*)-warfarin, anionic species were docked, while for quercetin and quercetin-3-*O*-glucuronide, two most prevalent anionic and fluorescent species [49] were docked. Afterward, ligands were read in the AutoDock software in a compatible file format and partial charges were set according to Ionescu et al. [50] As binding of flavonoids was confirmed to be at the Sudlow’s site I, grid maps of size 80 × 80 × 80 Å were generated with 0.375 Å spacing centered on the coordinates of the side-chain nitrogen in Trp-214 (3.466, −10.477, −4.189) in Sudlow’s site I by the AutoGrid program [47] and Lamarckian genetic algorithm (LGA) [51] was applied. The receptor molecule was regarded as rigid while all ligand single bonds were allowed to rotate freely during the Monte Carlo simulated annealing procedure. Ligand flexible docking simulations were performed with 100 runs, population size of 150, 2.5 × 10^7^ energy evaluations, 27,000 numbers of generations, rate of gene mutation of 0.02, and rate of crossover 0.08. Root-mean-square-deviation (rmsd) of 2.0 Å was used as a criterion for cluster analysis of the docking results (in order to determine if two docked conformations were similar enough to be included in the same cluster) and several such clusters with the lowest binding energy of each ligand species were used for further comparison.

## 4. Conclusions

Warfarin and selected flavonoid aglycons and glycosides do not share the same binding region as the IIA binding site. Rather, their binding regions are separated and loosely dependent of each other through allosteric modulation, i.e., conformational change of HSA or through a presence of warfarin LAS. While the warfarin binding region is located inside the hydrophobic pocket of the IIA subdomain, binding regions for flavonoid aglycons and glycosides are located at the hydrophilic entrance of the IIA binding pocket, where a putative warfarin LAS is also located. The only significant interaction between warfarin and flavonoid occurs at physiologically unattainable warfarin and flavonoid concentrations, but even at those concentrations, displacement is very questionable and can be due to energy transfer.

## Figures and Tables

**Table 1:** Investigated flavonoids and their binding constants: correlation between theoretical and experimental values.

Name	R_1_	R_2_	R_3_	*K* ± S.D. (× 10^4^ M^−1^)	Corr ^1^
Warfarin	N/A	N/A	N/A	10.56 ± 0.68	0.998
Luteolin	-H	-H	-OH	12.20 ± 0.53	0.999
Isoorientin	-H	-Glc	-OH	4.57 ± 0.30	0.999
Cynaroside	-H	-H	-*O*-Glc	4.20 ± 0.25	0.999
Quercetin	-OH	-H	-OH	14.40 ± 0.52	1.000
Isoquercitrin	-*O*-Glc	-H	-OH	4.47 ± 0.21	1.000
Hyperoside	-*O*-Gal	-H	-OH	3.24 ± 0.19	0.999
Quercetin-3-*O*-glucuronide	-*O*-Gluc	-H	-OH	5.36 ± 0.13	1.000
Rutin	-Glc-Rha	-H	-OH	2.92 ± 0.13	1.000

^1^ Correlation between experimental data and hypothesized 1:1 binding ratio calculated according to Equations (1)–(3). N/A: not applicable; S.D.: standard deviation.

**Table 2:** Summary of quercetin species docking.

**Quercetin Anion at Position 7**			**Quercetin Anion at Position 4’**		
**Cluster Rank**	**Number of Runs**	**Binding Energy (kcal/mol)**	**Cluster Rank**	**Number of Runs**	**Binding Energy (kcal/mol)**
1.	3	−4.79	1.	1	−4.68
2.	1	−4.42	2.	19	−4.38
3.	2	−4.22	3.	1	−4.30
4.	4	−4.20	4.	2	−4.16
5.	12	−4.12	5.	9	−4.11
6.	23	−4.07	6.	9	−4.08
7.	3	−3.94	7.	2	−3.69
8.	14	−3.94	8.	8	−3.68
**Fluorescent quercetin anion at positions 3 and 7**			**Fluorescent quercetin anion at positions 3 and 4’**		
**Cluster Rank**	**Number of Runs**	**Binding Energy (kcal/mol)**	**Cluster Rank**	**Number of Runs**	**Binding Energy (kcal/mol)**
1.	43	−4.66	1.	5	−4.42
2.	1	−4.61	2.	18	−4.04
3.	4	−4.60	3.	13	−3.87
4.	7	−4.24	4.	3	−3.82
5.	4	−4.20	5.	1	−3.81

**Table 3:** Summary of quercetin-3-*O*-glucuronide species docking.

**Quercetin-3-*O*-glucuronide Anion at Position 7**			**Quercetin-3-*O*-glucuronide Anion at Position 4’**		
**Cluster Rank**	**Number of Runs**	**Binding Energy (kcal/mol)**	**Cluster Rank**	**Number of Runs**	**Binding Energy (kcal/mol)**
1.	4	−5.02	1.	5	−6.09
2.	5	−4.76	2.	3	−4.89
3.	1	−4.62	3.	2	−4.78
4.	4	−4.49	4.	4	−4.74
5.	2	−4.49	5.	6	−4.52
6.	9	−4.45	6.	1	−4.16
7.	5	−4.45	7.	4	−4.02
8.	1	−4.35	8.	4	−3.99
**Fluorescent quercetin-3-*O*-glucuronide anion at position 7**			**Fluorescent quercetin-3-*O*-glucuronide anion at position 4’**		
**Cluster Rank**	**Number of Runs**	**Binding Energy (kcal/mol)**	**Cluster Rank**	**Number of Runs**	**Binding Energy (kcal/mol)**
1.	13	−4.21	1.	5	−4.42
2.	2	−4.07	2.	18	−4.04
3.	11	−4.06	3.	13	−3.87
4.	1	−4.05	4.	3	−3.82
5.	3	−3.95	5.	1	−3.81

**Table 4:** Summary of (*R*)- and (*S*)-warfarin low affinity site docking.

(*R*)-Warfarin			(*S*)-Warfarin		
Cluster Rank	Number of Runs	Binding Energy (kcal/mol)	Cluster Rank	Number of Runs	Binding Energy (kcal/mol)
1.	9	−6.70	1.	27	−6.70
2.	6	−6.52	2.	6	−6.56
3.	4	−6.49	3.	5	−6.51
4.	35	−6.28	4.	15	−6.42
5.	4	−6.27	5.	2	−6.31

## Supplementary Materials

Supplementary materials are available online.

## References

[1] Peters T. (1996). Ligand Binding by Albumin. All about Albumin.

[2] Peters T. (1996). Metabolism: Albumin in the Body. All about Albumin.

[3] Sudlow G., Birkett D.J., Wade D.N. (1975). Characterization of two specific drug binding sites on human serum albumin. Mol. Pharmacol..

[4] Chan E., McLachlan A.J., Pegg M., MacKay A.D., Cole R.B., Rowland M. (1994). Disposition of warfarin enantiomers and metabolites in patients during multiple dosing with rac-warfarin. Br. J. Clin. Pharmacol..

[5] Lee K., Woo H.I., Bang O.Y., On Y.K., Kim J.S., Lee S.Y. (2015). How to use warfarin assays in patient management: Analysis of 437 warfarin measurements in a clinical setting. Clin. Pharmacokinet..

[6] Jensen B.P., Chin P.K.L., Roberts R.L., Begg E.J. (2012). Influence of adult age on the total and free clearance and protein binding of (*R*)- and (*S*)-warfarin. Br. J. Clin. Pharmacol..

[7] Yacobi A., Udall J.A., Levy G. (1976). Serum protein binding as a determinant of warfarin body clearance and anticoagulant effect. Clin. Pharmacol. Ther..

[8] Diana F.J., Veronich K., Kapoor A.L. (1989). Binding of nonsteroidal anti-inflammatory agents and their effect on binding of racemic warfarin and its enantiomers to human serum albumin. J. Pharm. Sci..

[9] Kragh-Hansen U. (1981). Molecular aspects of ligand binding to serum albumin. Pharmacol. Rev..

[10] DeVane C.L. (2002). Clinical significance of drug binding, protein binding, and binding displacement drug interactions. Psychopharmacol. Bull..

[11] Hochman J., Tang C., Prueksaritanont T. (2015). Drug-drug interactions related to altered absorption and plasma protein binding: Theoretical and regulatory considerations, and an industry perspective. J. Pharm. Sci..

[12] Fanali G., Di Masi A., Trezza V., Marino M., Fasano M., Ascenzi P. (2012). Human serum albumin: From bench to bedside. Mol. Asp. Med..

[13] Benet L.Z., Hoener B.-A. (2002). Changes in plasma protein binding have little clinical relevance. Clin. Pharmacol. Ther..

[14] Pérez-Jiménez J., Fezeu L., Touvier M., Arnault N., Manach C., Hercberg S., Galan P., Scalbert A. (2011). Dietary intake of 337 polyphenols in French adults. Am. J. Clin. Nutr..

[15] Bors W., Michel C., Stettmaier K. (1997). Antioxidant effects of flavonoids. BioFactors.

[16] Dauchet L., Amouyel P., Hercberg S., Dallongeville J. (2006). Fruit and Vegetable Consumption and Risk of Coronary Heart Disease: A Meta-Analysis of Cohort Studies. J. Nutr..

[17] Bojić M., Debeljak Ž., Medić-Šarić M., Tomičić M. (2012). Interference of selected flavonoid aglycons in platelet aggregation assays. Clin. Chem. Lab. Med..

[18] Brglez Mojzer E., Knez Hrnčić M., Škerget M., Knez Ž., Bren U. (2016). Polyphenols: Extraction methods, antioxidative action, bioavailability and anticarcinogenic effects. Molecules.

[19] De Vries J.H.M., Hollman P.C.H., Meyboom S., Buysman M.N.C.P., Zock P.L., van Staveren W.A., Katan M.B. (1998). Plasma concentrations and urinary excretion of the antioxidant flavonols quercetin and kaempferol as biomarkers for dietary intake. Am. J. Clin. Nutr..

[20] Campanero M.A., Escolar M., Perez G., Garcia-Quetglas E., Sadaba B., Azanza J.R. (2010). Simultaneous determination of diosmin and diosmetin in human plasma by ion trap liquid chromatography-atmospheric pressure chemical ionization tandem mass spectrometry: Application to a clinical pharmacokinetic study. J. Pharm. Biomed. Anal..

[21] Dufour C., Dangles O. (2005). Flavonoid-serum albumin complexation: Determination of binding constants and binding sites by fluorescence spectroscopy. Biochim. Biophys. Acta Gen. Subj..

[22] Bi S., Ding L., Tian Y., Song D., Zhou X., Liu X., Zhang H. (2004). Investigation of the interaction between flavonoids and human serum albumin. J. Mol. Struct..

[23] Di Bari L., Ripoli S., Pradhan S., Salvadori P. (2010). Interactions between quercetin and warfarin for albumin binding: A new eye on food/drug interference. Chirality.

[24] Poór M., Li Y., Kunsági-Máté S., Petrik J., Vladimir-Knežević S., Koszegi T. (2013). Molecular displacement of warfarin from human serum albumin by flavonoid aglycones. J. Lumin..

[25] Graefe E.U., Wittig J., Mueller S., Riethling A.-K., Uehleke B., Drewelow B., Pforte H., Jacobasch G., Derendorf H., Veit M. (2001). Pharmacokinetics and bioavailability of quercetin glycosides in humans. Herb. Med..

[26] Dangles O., Dufour C., Manach C., Mornad C., Remesy C. (2001). Binding of flavonoids to plasma proteins. Method Enzymol..

[27] Boulton D.W., Walle U.K., Walle T. (1998). Extensive binding of the bioflavonoid quercetin to human plasma proteins. J. Pharmacol. Pharmacother..

[28] Zsila F., Bikádi Z., Simonyi M., Bika Z. (2003). Probing the binding of the flavonoid, quercetin to human serum albumin by circular dichroism, electronic absorption spectroscopy and molecular modelling methods. Biochem. Pharmacol..

[29] Epps D.E., Raub T.J., Caiolfa V., Chiari A., Zamai M. (1999). Determination of the affinity of drugs toward serum albumin by measurement of the quenching of the intrinsic tryptophan fluorescence of the protein. J. Pharm. Pharmacol..

[30] Eftink M.R., Ghiron C.A. (1981). Fluorescence quenching studies with proteins. Anal. Biochem..

[31] He X.M., Carter D.C. (1992). Atomic structure and chemistry of human serum albumin. Nature.

[32] Wolfbeis O.S., Begum M., Geiger H. (1984). Fluorescence properties of hydroxy- and methoxyflavones and the effect of shift reagents. Z. Für Naturforsch..

[33] Peters T. (1996). The Albumin molecule: Its structure and chemical properites. All about Albumin.

[34] D’Arcy P.F., McElnay J.C. (1982). Drug interactions involving the displacement of drugs from plasma protein and tissue binding sites. Pharmacol. Ther..

[35] Sjöholm I., Ekman B., Kober A., Ljungstedt-Påhlman I., Seiving B., Sjödin T. (1979). Binding of drugs to human serum albumin: XI. The specificity of three binding sites as studied with albumin immobilized in microparticles. Mol. Pharmacol..

[36] Schmidt S., Gonzales D., Derendorf H. (2010). Significance of protein binding in pharmacokinetics and pharmacodynamics. J. Pharm. Sci..

[37] Lagercrantz C., Larsson T., Denfors I. (1981). Stereoselective binding of the enantiomers of warfarin and trytophan to serum albumin from some different species studied by affinity chromatography on columns of immobilized serum albumin. Comp. Biochem. Physiol. Part C Comp. Pharmacol..

[38] Petitpas I., Bhattacharya A.A., Twine S., East M., Curry S. (2001). Crystal structure analysis of warfarin binding to human serum albumin. Anatomy of drug site I. J. Biol. Chem..

[39] Ni Y., Zhang X., Kokot S. (2009). Spectrometric and voltammetric studies of the interaction between quercetin and bovine serum albumin using warfarin as site marker with the aid of chemometrics. Spectrochim. Acta Part A Mol. Biomol. Spectrosc..

[40] Bren U., Oostenbrink C. (2012). Cytochrome P450 3A4 inhibition by ketoconazole: Tackling the problem of ligand cooperativity using molecular dynamics simulations and free-energy calculations. J. Chem. Inf. Model..

[41] Guharay J., Sengupta B., Sengupta P.K. (2001). Protein-flavonol interaction: Fluorescence spectroscopic study. Proteins Struct. Funct. Genet..

[42] Omidvar Z., Parivar K., Sanee H., Amiri-Tehranizadeh Z., Baratian A., Saberi M.R., Asoodeh A., Chamani J. (2011). Investigations with spectroscopy, zeta potential and molecular modeling of the non-cooperative behaviour between cyclophosphamide hydrochloride and aspirin upon interaction with human serum albumin: Binary and ternary systems from multi-drug therapy. J. Biomol. Struct. Dyn..

[43] Yamasaki K., Maruyama T., Kragh-Hansen U., Otagiri M. (1996). Characterization of site I on human serum albumin: Concept about the structure of a drug binding site. Biochim. Biophys. Acta Protein Struct. Mol. Enzymol..

[44] Ghuman J., Zunszain P.A., Petitpas I., Bhattacharya A.A., Otagiri M., Curry S. (2005). Structural basis of the drug-binding specificity of human serum albumin. J. Mol. Biol..

[45] Yamasaki K., Maruyama T., Takadate A., Suenaga A., Kragh-Hansen U., Otagiri M. (2004). Characterization of site I of human serum albumin using spectroscopic analyses: Locational relations between regions Ib and Ic of site I. J. Pharm. Sci..

[46] Poór M., Boda G., Needs P.W., Kroon P.A., Lemli B., Bencsik T. (2017). Interaction of quercetin and its metabolites with warfarin: Displacement of warfarin from serum albumin and inhibition of CYP2C9 enzyme. Biomed. Pharmacother..

[47] Morris G.M., Huey R., Lindstrom W., Sanner M.F., Belew R.K., Goodsell D.S., Olson A.J. (2009). AutoDock4 and AutoDockTools4: Automated docking with selective receptor flexibility. J. Comput. Chem..

[48] Rimac H., Debeljak Ž., Šakić D., Weitner T., Gabričević M., Vrček V., Zorc B., Bojić M. (2016). Structural and electronic determinants of flavonoid binding to human serum albumin: An extensive ligand-based study. RSC Adv..

[49] Dangles O., Dufour C., Bret S. (1999). Flavonol-serum albumin complexation. Two-electron oxidation of flavonols and their complexes with serum albumin. J. Chem. Soc. Perkin Trans..

[50] Ionescu C.-M., Sehnal D., Falginella F.L., Pant P., Pravda L., Bouchal T. (2015). AtomicChargeCalculator: Interactive web-based calculation of atomic charges in large biomolecular complexes and drug-like molecules. J. Cheminform..

[51] Huey R., Morris G.M., Olson A.J., Goodsell D.S. (2007). A semiempirical free energy force field with charge-based desolvation. J. Comput. Chem..

