# Supplementary material for: Warfarin and Flavonoids Do Not Share the Same Binding Region in Binding to the IIA Subdomain of Human Serum Albumin
Source: Molecules. 2017 Jul 11;22(7):1153. doi: 10.3390/molecules22071153 (PMC6152318; doi:10.3390/molecules22071153)
Supplement: Supplementary file 1 [file molecules-22-01153-s001.pdf]

Supplementary file

Warfarin and flavonoids do not share the same binding region in binding to the IIA subdomain of human serum albumin

Hrvoje Rimac, Claire Dufour, Željko Debeljak, Branka Zorc, and Mirza Bojić

Table S1. Dependency of fluorescence on warfarin concentration at wavelengths of excitation and emission wavelengths of 317 and 380 nm, respectively.

Warfarin concentration [µM]	0% occupied HSA	63% occupied HSA
0,00	0%	0%
0,40	3%	2%
0,80	5%	5%
1,20	8%	7%
2,00	12%	10%
2,99	18%	17%
3,98	23%	21%
5,96	33%	30%
7,92	43%	39%
9,88	50%	47%
13,76	64%	61%
17,60	75%	72%
21,41	83%	80%
27,05	93%	91%
33,72	98%	96%
41,71	100%	97%

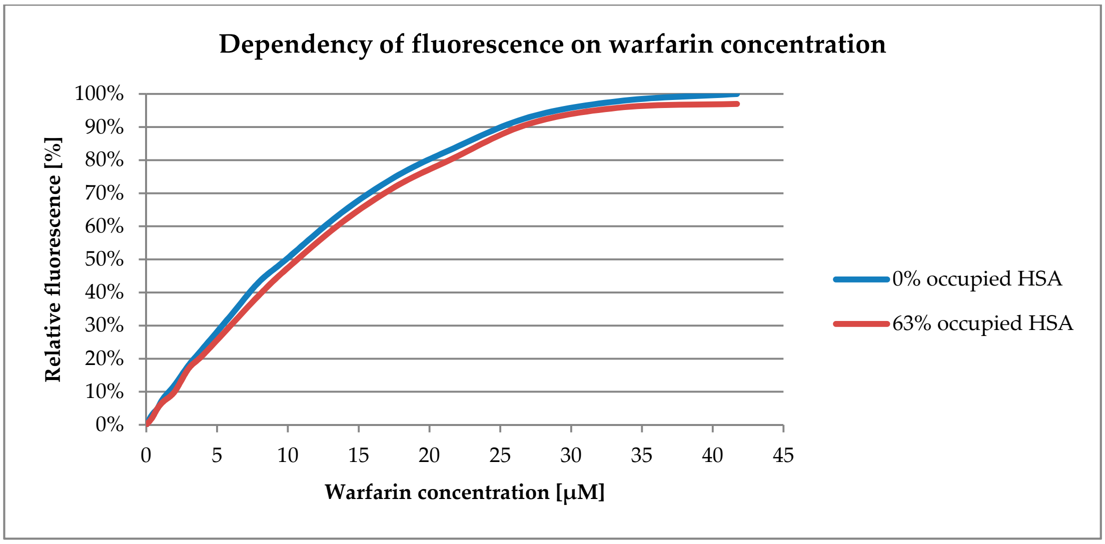

Figure S1. Relative fluorescence versus warfarin concentration for 0% (blue) and 63% occupied HSA (red).

Table S2. Absolute fluorescence intensities of (**A**) quercetin and (**B**) luteolin.

(**A**)

Quercetin concentration [uM]	Buffer solution	0% occupied HSA	22% occupied HSA	38% occupied HSA	63% occupied HSA	92% occupied HSA
0,00	0,6147	1,1810	1,4770	1,7299	1,4261	1,1932
2,00	0,8077	14,1006	14,3985	15,6001	14,9774	12,2760
4,00	0,9540	18,1924	19,1798	20,1073	20,0687	16,0796
6,00		21,7864	22,8342	23,2658	23,0451	18,9492
7,99	1,1279	25,2012	26,8970	26,0412	25,4057	21,2821
11,98		30,3340	31,0510	30,0611	29,3172	25,1156
15,97	1,3080	33,9655	33,9646	33,4123	32,2916	28,2109
23,93		38,4893	37,6942	37,3757	36,2415	32,2392
31,87	1,5574	40,5581	39,7022	39,4192	37,9878	34,7552
39,80		**41,9281**	41,0854	41,0246	39,4685	36,6753
47,71	1,7477	41,6617	41,5821	41,3733	40,4449	37,3158
63,49	2,0820	40,9892	40,4660	40,7086	39,7702	38,0617
79,21	2,2472	39,3310	39,3997	39,3849	38,8686	37,3045
98,77	2,4600	37,4633	37,3641	37,6865	37,3324	36,3823

(**B**)

Luteolin concentration [uM]	Buffer solution	0% occupied HSA	22% occupied HSA	38% occupied HSA	63% occupied HSA	92% occupied HSA
0,00	0,5716	1,0372	1,1877	1,0684	1,2011	1,1386
2,00	0,5984	3,5310	3,0516	3,2663	2,9688	3,0838
4,00	0,7341	5,6541	5,0287	5,0961	4,7527	4,7445
6,00		7,6599	6,8528	6,8106	6,3873	6,5159
7,99	0,8094	9,4059	8,8217	8,5002	7,8443	8,1746
11,98		11,5397	11,3861	11,3711	10,9648	10,4774
15,97	0,8481	14,0865	14,1260	13,9507	13,0693	12,9194
23,93		17,9442	17,8085	17,8530	17,0150	16,3518
31,87	1,0538	20,8667	20,4964	20,5131	19,6701	19,1278
39,80		22,9301	22,5924	22,2082	21,6524	20,8642
47,71	1,3400	24,5206	23,8063	23,5737	23,1678	22,5713
63,49	1,5045	26,4505	25,9034	25,4318	24,8572	24,6423
79,21	1,7483	26,9488	26,5002	26,5136	25,8191	25,3909
98,77		**27,2939**	27,2804	26,9116	26,1311	26,2198
118,23	2,2774	27,1078	27,1338	26,9198	26,3501	26,3929
137,59		26,3217	26,7053	26,6416	26,1054	26,3918
156,86	2,8851	25,8093	26,0626	26,1509	25,5239	25,7773
